# Diarrhoea and acute respiratory infections prevalence and risk factors among under-five children in Iraq in 2000

**DOI:** 10.1186/1824-7288-35-8

**Published:** 2009-04-25

**Authors:** Seter Siziya, Adamson S Muula, Emmanuel Rudatsikira

**Affiliations:** 1 Department of Community Medicine, University of Zambia, School of Medicine, Lusaka, Zambia; 2 Department of Community Health, College of Medicine, University of Malawi, Blantyre, Malawi; 3 Division of Epidemiology and Biostatistics, San Diego State University, San Diego, California, USA

## Abstract

**Background:**

Diarrhoea and acute respiratory conditions are common medical conditions among under-five children in resource-limited and conflict situations. The present study was conducted to estimate the prevalence and associated factors for acute respiratory conditions and diarrhoea among children under the age of five years in Iraq in 2000.

**Methods:**

Data for the Iraqi Multiple Indicator Cluster Survey were obtained from UNICEF. We estimated the prevalence of acute respiratory conditions and diarrhoea. Assessment of the associations between these two medical conditions on one hand and socio-demographic and environmental variables on the other was done using logistic regression analysis. Weighted analysis was conducted to account for complex survey design.

**Results:**

A total of 14,676 children under the age of 5 years were reported by their mothers in the study. Of these 50.4% were males. About half (53.9%) of the children had complete vaccination status. Overall, 21.3% of the children had diarrhoea, and 6.9% had acute respiratory infection (ARI) in the last two weeks. In multivariate analysis, diarrhoea was associated with age of child, area of residence, maternal education, source of water, toilet facility, disposal of children' stool and disposal of dirty water. Compared to children aged 48–59 months, children in the age groups 6–11 months and 12–23 months were 2.22 (95%CI [2.02, 2.44]) and 1.84 (95%CI [1.71, 2.00]) times more likely, respectively, to have diarrhoea. Children whose mothers had no formal education were 11% (AOR = 1.11, 95%CI [1.04, 1.18]) more likely to have diarrhoea compared to children with mothers who had attained secondary level of education. Compared to children who belonged to households with unprotected well or river as the main source of water, children who belonged to households with piped water were 32% (AOR = 1.32, 95%CI [1.17, 1.48]) more likely to have diarrhoea while those who belonged to households with protected well were 26% (AOR = 0.74, 95%CI [0.62, 0.89]) less likely to have diarrhoea. Age of child, toilet facility, wealth, and sex of child were significantly associated with ARI.

**Conclusion:**

In a study of under-five children in Iraq in 2000, we found that history of diarrhoea and ARI were negatively associated with lower socio-economic status, adequate disposal of children's stool and dirty water, but the results were inconsistent in terms of access to potable water and sanitation facilities possibly due to non-functioning of water and sewage plants after the war. Improvement in water quality and sanitation are vital in the reduction of diarrhoeal diseases.

## Background

Diarrhoea and respiratory diseases are major causes of child mortality in low and middle-income countries [1,2]. Much of the published literature on global childhood diarrhoea and respiratory conditions however has been focused on Africa, South East Asia and the Indian sub-continent. Fewer reports have been published from the Middle Eastern region where, as was the situation in Iraq during the sanction years following the 1991 Gulf war, social and economic conditions deteriorated significantly.

The health status of children in Iraq deteriorated starting from the end of the (First) Gulf War in 1991 well past the beginning of the 21^st^century, with increased mortality among children of age 5 years and younger [3-5]. However, mortality estimates have not been consistent among Iraqi children as reported by various researchers and program agencies. Of particular mention are the reports by Blacker et al in 2003 [3], who reported that the number of deaths of children under 5 in Iraq in the period 1991–98 resulting from the Gulf War of 1991 and the subsequent imposition of comprehensive sanctions by the United Nations were to lie, with 95% certainty between 380,000 and 480,000. Mason et al [4], however, estimated that in the period 1991–96 the number of excess under-5 deaths was no more than 68,000. Blacker et al [3,5] have attempted to explain the discrepancies in the mortality estimates by suggesting that different sources of data and assumptions for analysis were the main reasons for the inconsistencies in estimates. The sources of data varied from demographic surveys, hospital based data, nutritional assessment surveys, to government and independent reports. Assumptions that were considered in some estimates included: average mortality rates prior to the war remained constant during and after the war; and mortality had continued to decline at the rate observed between 1974 and 1990.

In terms of rates, Tawfeek et al [6] reported a mortality rate of 28.6% among under-five children presenting to Baghdad hospitals between 1990 and 1997. All hospitals experienced serious problems with lighting, cleaning, water supply and sewage; drug supplies and operations; and half of all diagnostic and therapeutic equipment was not working due to lack of spare parts [7] despite the fact that medical supplies were not part of the sanctions [8]. Dobson [9] estimates that the mortality rate for under-five children increased from 56 per 1000 live births in the period 1984–89 before the sanctions to 131 per 1000 live births in the period 1994–99. He attributed this increase to economic collapse, poor sanitation, lack of safe water, and inadequate provision of health care. Similar findings were reported for the period 1991–98 by UNICEF who indicated that countrywide, 131 deaths per 1000 live births occurred among under-five children compared to 40 per 1000 in 1989 [10].

The importance of diarrhoea and acute respiratory infections among children cannot be underestimated. Garfield [11] reports that the increase in mortality accounting for 100,000–227,000 excess deaths among children under-five years of age in the period 1990–98 was caused mainly by diarrhoea and respiratory illnesses. He suggested that the underlying causes included contaminated water and inadequate supplies in the curative health care system.

While a report and discussion on the estimated infant and under-5 mortality in Iraq is a worthwhile exercise in its own right, our current study aimed to explore the factors that were associated with reported diarrhoea and respiratory conditions among children in Iraq using data from the Iraq Multiple Cluster Sampling Survey 2000. This information would be useful to identify children at risk of diarrhoea and acute respiratory infections living under difficult circumstances such as economic, social, human rights and personal safety crises. Our study has, therefore, potential to inform interventions as to which children are particularly vulnerable for diarrhoea and acute respiratory infections in difficult post-war and economic sanctions period.

## Methods

### Source of data and survey description

The present study was exempted from institutional review board assessment because secondary data obtained from UNICEF were used. The data were from the 2000 Iraqi Multiple Indicator Cluster Survey. A complete description of this survey has been reported before [12]. However in summary, this survey was designed to be representative of the whole country and covered all the 18 governorates of Iraq. Iraq is divided into Governorates, then into *Qada'as *(districts) that are further divided into sub-districts called *Nahiya*. These sub-districts are further divided into Quarters called *Mahala *in urban areas and villages in rural areas.

Three stages of sampling were conducted as follows: in the first stage a number of *Mahalas *(in urban areas) or villages (in rural areas) in each *Qada'a *were selected according to probability proportional to the size of a *Qada'a*. During the second stage of sampling, a *Mahala *or village was divided into segments with a population of approximately 500 each. One segment or more was selected with probability proportionate to the size of a *Mahala *or village. Furthermore, each segment was divided into blocks or *Majals *with 25–30 households in urban areas and 20–25 households in rural areas. One *Majal *was subsequently selected using a simple random sampling method. In the final stage of sampling, a listing of households in each selected *Majal *was carried out, and a cluster of 10 households was selected using a systematic random sampling technique.

### Questionnaires and survey administration

The questionnaire used in the 2000 Iraqi MICS was modeled from the UNICEF's Middle Eastern and North African Regional Office (MENARO) core MICS questionnaire. The questionnaire was translated into Arabic with some revisions and adaptations. Data were collected regarding household socio-demographic characteristics, maternal education, access to potable water and sanitation. A total of 14,744 children under five years of age were eligible to be included in the survey, however, mothers of 14,676 children were eventually interviewed, giving a response rate of 99.5% overall; 99.6% and 99.5% for the urban and rural areas, respectively.

### Variables

Two outcome variables were included in this study. These were: history of diarrhoea in the past 2 weeks (Yes/No) and symptoms and signs of acute respiratory infections (Yes/no). The question for diarrhoea was: Has (child's *name*) had diarrhea in the last two weeks? Diarrhoea was defined as perceived by mother or caretaker, or as three or more loose or watery stools per day, or blood in stool. Acute respiratory infection was defined as a report of a child whose parent reported yes to all of the following questions: In the last two weeks, has <name of child> had any other illness, such as cough or fever, or any other health problem? Has <name of child> had an illness with a cough at any time in the last two weeks, that is, since <day of the week> of the week before last? When <name> had an illness with a cough, did he/she breathe faster than usual with short, quick breaths or have difficulty breathing? Were the symptoms due to a problem in the chest or a blocked nose? Potentially associated variables included: child-related (age, sex, vaccination, breastfeeding history); maternal (formal education); and household (access to safe water, sanitation, wealth and crowding index) factors.

### Vaccinations

A child was deemed to have received full vaccination if he or she had all Expanded Programme on Immunizations (EPI) vaccines at appropriate age which in Iraq included: BCG in the first week of life; DPT and OPV first dose at 2 months of age; DPT and OPV second dose at 4 months of age; DPT and OPV third dose at 6 months of age; Measles at 9 months of age; Measles, Mumps and Rubella at 15 months of age; and DPT and OPV first booster 1 year after last dose [13].

### Wealth index

Household wealth was defined based on household assets reported by the survey participant. Each asset was assigned a weighting value, using principal component analysis as described by the World Bank [14]. A household was assigned a standardized score for each owned asset. For each household, these scores were summed and households ranked into five wealth quintiles i.e. the lowest quintile being the poorest and the highest quintile the least poor.

### Crowding index

The density or crowding in the home was assessed using a crowding index defined as the total number of family members in the home divided by the total number of rooms in the home, excluding the kitchen and bathroom [15]. The crowding index was scored as low (if 0–1 co-residents), moderate (scores 2–3 co-residents), or high (score > 3 co-residents) per room.

### Data analysis

Weighted analysis using SPSS version 11.5 (SPSS, Chicago, Illinois, United States) was conducted to account for the complex survey design. We calculated the frequencies and proportions of outcome variables (diarrhoea in the past two weeks and a respiratory condition in the past two weeks). Variables statistically significant at bivariate analyses were considered as potential confounders in a multivariate logistic regression analysis. The α-level was set at 0.05.

## Results

### Socio-demographic characteristics of the children in the 2000 Iraqi MICS

The number of children whose data were available was 14,587 of the 14,676 reported to have participated. Of these 50.4% were males. Overall, 20.8% of the children were of age less than one year. Only 25.3% of the mothers had attained secondary level of education, and 36.4% of the participants lived in rural areas. About a quarter (25.3%) of the participants belonged to the poorest wealth index quintile. The majority (96.2%) of the children had ever been breastfed. Most children belonged to households that had piped water (80.2%), flush toilets (73.1%), and disposed off children's stool in toilet (74.0%). About half (53.9%) of the children had complete vaccination status. Overall, 21.3% of the children had diarrhoea, and 6.9% had acute respiratory infection (ARI) in the previous two weeks. Further description of the sample with regard to socio-economic variables is shown in Table 1.

**Table 1:** Socio-demographic characteristics of the children in the Iraq MICS 2000

	**Total**	**Male**	**Female**
**Characteristic**	n* (%)**	n* (%)**	n* (%)**
Sex of child	14587 (100)	7393 (50.4)	7194 (49.6)
Age of child			
<6	1327 (9.5)	676 (9.0)	651 (9.9)
6–11	1667 (11.3)	858 (11.4)	809 (11.2)
12–23	3009 (20.3)	1466 (19.7)	1543 (21.0)
24–35	2766 (18.8)	1420 (18.8)	1346 (18.8)
36–47	3133 (21.7)	1551 (21.6)	1582 (21.7)
48–59	2685 (18.4)	1422 (19.4)	1263 (17.4)
Maternal formal education			
None	5551 (33.6)	2759 (33.0)	2792 (34.1)
Primary	5868 (41.1)	3029 (42.0)	2839 (40.2)
Secondary	3163 (25.3)	1603 (24.9)	1560 (25.7)
Area of residence			
Urban	7765 (63.6)	3939 (63.3)	3826 (64.0)
Rural	6822 (36.4)	3454 (36.7)	3368 (36.0)
Crowding index			
≤ 2.00	6278 (46.8)	3243 (47.5)	3035 (46.1)
2.01–3.00	4588 (30.1)	2321 (29.6)	2267 (30.5)
>3.00	3696 (23.1)	1818 (22.9)	1878 (23.4)
Wealth index (quintiles)			
Poorest	4832 (25.3)	2473 (25.8)	2359 (24.9)
Second	3060 (21.9)	1520 (21.5)	1540 (22.2)
Middle	2822 (19.9)	1440 (20.3)	1382 (19.6)
Fourth	1980 (20.0)	989 (19.4)	991 (20.6)
Richest	1868 (12.9)	960 (13.0)	908 (12.7)
Ever breastfed			
No	487 (3.8)	250 (3.8)	237 (3.9)
Yes	14089 (96.2)	7140 (96.2)	6949 (96.1)
Main source of water			
Piped	10532 (80.2)	5346 (80.1)	5186 (80.3)
Protected well	637 (4.2)	326 (4.3)	311 (4.2)
Unprotected well/river	3393 (15.6)	1710 (15.7)	1683 (15.5)
Toilet facility			
Flush toilet	9341 (73.1)	4728 (72.9)	4613 (73.4)
Improved pit latrine	816 (4.2)	419 (4.3)	397 (4.2)
Traditional pit latrine	2515 (13.8)	1283 (14.2)	1232 (13.5)
Open pit	421 (2.1)	211 (2.1)	210 (2.1)
No facility, bush	1427 (6.7)	721 (6.7)	706 (6.7)
Disposal of children's stool			
Thrown in toilet/pit latrine	9124 (74.0)	4649 (74.3)	4475 (73.6)
Thrown outside the yard	3932 (22.0)	1942 (21.5)	1990 (22.5)
Left on the ground	769 (4.0)	412 (4.2)	357 (3.8)
Disposal of dirty water			
Net joining public sewer system	1085 (16.7)	541 (16.4)	544 (16.9)
Net that inadequately disposes dirty water	1442 (10.1)	724 (10.0)	718 (10.1)
Net joining septic tank	7571 (51.0)	3855 (51.0)	3716 (50.9)
Water poured to form collections	4302 (22.3)	2174 (22.6)	2128 (22.0)
Vaccination status			
Incomplete	7084 (46.1)	3570 (45.6)	3514 (46.5)
Complete	7503 (53.9)	3823 (54.4)	3680 (53.5)
Diarrhoea			
No	11594 (78.7)	5835 (78.1)	5759 (79.3)
Yes	2993 (21.3)	1558 (21.9)	1435 (20.7)
Acute respiratory infection			
No	13604 (93.1)	6817 (92.2)	6787 (94.1)
Yes	983 (6.9)	576 (7.8)	407 (5.9)

* unweighted frequency ** weighted percent

### Correlates for diarrhoea, and acute respiratory infection

In bivariate logistic analysis, factors associated with diarrhoea and ARI were age of child, wealth, main source of water, toilet facility, and disposal of dirty water. Meanwhile, factors associated with diarrhoea alone were maternal education, area of residence, disposal of children's stool, and vaccination status. Child's gender was associated with only ARI. Lastly, the factors ever breastfed and crowding were not significantly associated with both diarrhoea, and ARI. These results are shown in Table 2.

**Table 2:** Correlates for diarrhoea, and acute respiratory infection among children aged less than 5 years in bivariate analyses

	Unadjusted OR* (95%CI)	
**Characteristic**	**Diarrhoea**	**Acute respiratory infection**
Sex of child		
Male	1.04 (0.99, 1.08)	1.17 (1.09, 1.24)
Female	1	1
Age of child		
<6	0.98 (0.87, 1.10)	0.76 (0.62, 0.93)
6–11	2.20 (2.01, 2.42)	1.49 (1.28, 1.73)
12–23	1.83 (1.70, 1.98)	1.30 (1.14, 1.47)
24–35	0.95 (0.87, 1.03)	1.14 (0.99, 1.30)
36–47	0.59 (0.53, 0.64)	0.82 (0.71, 0.95)
48–59	1	1
Maternal formal education		
None	1.00 (0.95, 1.06)	1.03 (0.94, 1.13)
Primary	1.08 (1.02, 1.14)	1.08 (0.99, 1.19)
Secondary	1	1
Area of residence		
Urban	1.12 (1.08, 1.17)	0.99 (0.93, 1.06)
Rural	1	1
Crowding index		
≤ 2.00	0.95 (0.90, 1.01)	0.98 (0.90, 1.07)
2.01–3.00	1.00 (0.95, 1.06)	1.06 (0.96, 1.16)
>3.00	1	1
Wealth index (quintiles)		
Poorest	0.79 (0.73, 0.85)	0.71 (0.62, 0.81)
Second	1.13 (1.05, 1.22)	1.50 (1.34, 1.68)
Middle	1.13 (1.05, 1.22)	1.23 (1.08, 1.39)
Fourth	0.96 (0.89, 1.04)	0.84 (0.73, 0.97)
Richest	1	1
Ever breastfed		
No	1.01 (0.91, 1.12)	1.00 (0.84, 1.18)
Yes	1	1
Main source of water		
Piped	1.36 (1.24, 1.49)	1.23 (1.07, 1.43)
Protected well	0.72 (0.62, 0.85)	0.78 (0.60, 1.01)
Unprotected well/river	1	1
Toilet facility		
Flush toilet	1.29 (1.18, 1.41)	1.42 (1.22, 1.67)
Improved pit latrine	0.89 (0.74, 1.07)	1.10 (0.82, 1.49)
Traditional pit latrine	0.92 (0.81, 1.04)	0.92 (0.74, 1.14)
Open pit	1.02 (0.80, 1.29)	0.83 (0.54, 1.29)
No facility, bush	1	1
Disposal of children's stool		
Thrown in toilet/pit latrine	1.03 (0.95, 1.12)	0.98 (0.87, 1.11)
Thrown outside the yard	0.90 (0.83, 0.99)	0.92 (0.80, 1.06)
Left on the ground	1	1
Disposal of dirty water		
Net joining public sewer system	0.93 (0.85, 1.01)	0.91 (0.79, 1.04)
Net that inadequately disposes dirty water	1.16 (1.05, 1.28)	1.22 (1.05, 1.42)
Net joining septic tank	1.15 (1.09, 1.23)	1.13 (1.03, 1.25)
Water poured to form collections	1	1
Vaccination status		
Incomplete	1.09(1.04, 1.13)	0.95(0.89, 1.02)
Complete	1	1

### Independent factors associated with diarrhoea, and acute respiratory infection

The following factors were associated with diarrhoea in multivariate analysis: age of child, maternal education, main source of water, toilet facility, area of residence, disposal of children's stool, and disposal of dirty water (Table 3). Compared to children aged 48–59 months, children in the age groups 6–11 months and 12–23 months were 2.22 (95%CI [2.02, 2.44]) and 1.84 (95%CI [1.71, 2.00]) times more likely, respectively, to have diarrhoea. Children of mothers with no formal education were 11% (AOR = 1.11, 95%CI [1.04, 1.18]) more likely to have diarrhoea compared to children with mothers who had attained secondary level of education.

**Table 3:** Correlates for diarrhoea, and acute respiratory infection among children aged less than 5 years in multivariate analysis

	Adjusted OR* (95%CI)	
**Characteristic**	**Diarrhoea**	**Acute respiratory infection**
Sex of child		
Male	-	1.17 (1.10, 1.25)
Female		1
		
Age of child		
<6	0.97 (0.86, 1.09)	0.76 (0.61, 0.93)
6–11	2.22 (2.02, 2.44)	1.49 (1.26, 1.74)
12–23	1.84 (1.71, 2.00)	1.32 (1.16, 1.49)
24–35	0.94 (0.85, 1.02)	1.15 (1.00, 1.31)
36–47	0.60 (0.55, 0.66)	0.83 (0.71, 0.95)
48–59	1	1
		
Area of residence		
Urban	1.08 (1.02, 1.15)	-
Rural	1	
		
Maternal formal education		
None	1.11 (1.04, 1.18)	-
Primary	1.05 (0.99, 1.11)	
Secondary	1	
		
Wealth index (quintiles)		
Poorest	-	0.89 (0.72, 1.12)
Second		1.51 (1.35, 1.70)
Middle		1.13 (0.98, 1.29)
Fourth		0.78 (0.68, 0.91)
Richest		1
		
Main source of water		
Piped	1.32 (1.17, 1.48)	-
Protected well	0.74 (0.62, 0.89)	
Unprotected well/river	1	
		
Toilet facility		
Flush toilet	1.37 (1.17, 1.48)	1.35 (1.07, 1.69)
Improved pit latrine	0.95 (0.78, 1.16)	1.07 (0.79, 1.45)
Traditional pit latrine	0.93 (0.81, 1.06)	0.89 (0.72, 1.11)
Open pit	0.98 (0.76, 1.27)	0.79 (0.51, 1.24)
No facility, bush	1	1
		
Disposal of children's stool		
Thrown in toilet/pit latrine	0.80 (0.72, 0.90)	-
Thrown outside the yard	0.92 (0.83, 1.01)	
Left on the ground	1	
		
Disposal of dirty water		
Net joining public sewer system	0.85 (0.76, 0.94)	-
Net that inadequately disposes dirty water	1.15 (1.03, 1.28)	
Net joining septic tank	1.00 (0.93, 1.09)	
Water poured to form collections	1	

Compared to children who belonged to households that had a network joining with unprotected wells or rivers as the main source of water, children who belonged to households with piped water were 32% (AOR = 1.32, 95%CI [1.17, 1.48]) more likely to have diarrhoea, and those who belonged to households with protected wells were 26% (AOR = 0.74, 95%CI [0.62, 0.89]) less likely to have diarrhoea. Children from households with flush toilets were 37% (AOR = 1.37, 95%CI [1.17, 1.48]) more likely to have diarrhoea compared with children from households that had no toilet facilities.

Compared to children who were from households that left children's stool on the ground, children from households that put children's stool in the toilet were 20% (AOR = 0.80, 95%CI [0.72, 0.90]) less likely to have diarrhoea. Children from households that had a net joining a public sewage system as a means of disposing dirty water were 15% (AOR = 0.85, 95%CI [0.76, 0.94]) less likely to have diarrhoea, compared to children from households that poured dirty water on the ground to form collections. Children from urban areas were 8% (AOR = 1.08, 95%CI [1.02, 1.15]) more likely to have diarrhoea compared to children from rural areas.

Age of child, toilet facility, wealth, and sex of child were significantly associated with ARI. While children aged below 6 months, and those in the age group 36–47 months were less likely to have ARI, children between the age of 6 and 35 months were more likely to have ARI compared to children in the age group 48–59 months. Compared to children from households that had no toilet facility, children from households that had flush toilets were 35% (AOR = 1.35, 95%CI [1.07, 1.69] more likely to have ARI. Children who belonged to the second and fourth wealth quintiles were less likely, respectively, to have ARI compared to children who belonged to the richest quintile. A male child was 17% (AOR = 1.17, 95%CI [1.10, 1.25] more likely to have ARI compared to a female child.

## Discussion

In a nationally representative survey of Iraqi women 15 to 49 years who had children under the age of 5 years, 6.9% and 21.3% of children enrolled in the survey were reported to have had ARI and diarrhoea, respectively, within the past 2 weeks. In multivariate logistic regression analysis, diarrhoea was positively associated with urban residence, ages 6 to 23 months, lower maternal education, having piped water and flush toilet, and having a network that inadequately disposed dirty water. ARI was associated with child age between 6 and 23 months, males, being in a household within 2^nd ^wealth quintile and having flush toilet facilities.

The Gulf War of 1991 put most pumping and sewage treatment plants out of action thereby discharging raw sewer into rivers, contaminating domestic water supply, and leading to high diarrhoea prevalence [7,9,14,15]. Furthermore, there are reports of women receiving from government free but limited supplies of formula milk in the absence of breastfeeding [16]. Malnutrition was not considered a major public health problem before the war in 1991 but since then the prevalence of chronic malnutrition increased from 18% to 31% in 1996 among under-five children in Iraq [17]. Aal-Kubaisy et al [18] in 2002 reported prevalence of xerophthalmia of 29%, among children aged <6 years admitted to medical wards for a variety of medical conditions, suggesting nutritional inadequacies during this time. The underlying malnutrition may have exposed children to diarrheal episodes.

Under the Iraqi culture, just like in most developing countries, children are the mother's responsibilities, even when a mother may work outside the home; she retains all child-care responsibilities [19]. Women who had limited education in our study may have also been less likely to adhere to proper handling of food. The better educated mothers may have been more likely to boil drinking water for their young children; thus reducing their children' exposure to pathogens. Significant associations between having a mother with little or no education and diarrhoea have been reported elsewhere [20-23].

Women who reported living in households with their main water supply as piped water and had homes with flush toilet were more likely to have children who experienced diarrhoea, while women who used protected wells were less likely to have children who had diarrhoea. As a result of the Gulf war and sanctions, water treatment plants lacked spare parts, equipment, treatment chemicals, proper maintenance and adequately qualified staff such that the plants acted solely as pumping stations without treatment [17]. Moreover, the water distribution network on which most of the population relied on had been destroyed, blocked or had leaky pipes. If water supply was erratic such that families had to store their water, this stored water if contaminated can transmit diarrhoea-causing pathogens. Similarly, flush toilets are effective if houses receive water 24 hours a day. Similar observations were made by Etiler et al [20] who reported a significant association between unhygienic toilet and diarrhoea, and by Rahmanifar et al [24] who found that poor household sanitation was associated with diarrhoeal illnesses in an Egyptian village.

We found that male children were more likely to have been reported as having had respiratory condition in the past 2 weeks than females. This is probably not surprising noting that where male-female differences have been observed in terms of child health, males almost always have had poor outcomes compared to females. Children in the age range 6–35 months were also likely to have had respiratory conditions in the past 2 weeks compared to other age groups. This age group, 6–35 months, could be the age group when children had not developed protective immunity but maternally acquired (passive) immunity was waning.

We expected that children from households with flush toilets would have had lower likelihood of having ARI. However, we found that they had higher odds of ARI than those from households with no toilet facilities. We can again suggest that with erratic water supplies during the period surrounding the year 2000, the effectiveness of flush toilets at least in preventing diarrheal may have been compromised. Finally, the lower odds of ARI among children in households in the poorest wealth quintile, is unexpected, if one compares this group to the second wealth quintile. Furthermore, Sha et al [25] found children in low and medium social economic status (SES) groups to have had higher respiratory morbidity than those in the high SES group. We can speculate that due to survival bias, more children in the poorest quintile than in any other quintile could have died, resulting in similar odds of ARI in both the poorest and richest quintiles.

Despite the fact that the 2000 Iraq MICS was a nationally representative sample and had a large sample size, the current study has a number of limitations. Firstly, data collection was cross sectional. This, therefore, means that we can not assign causation on the association between any explanatory variable and the outcomes of interest. Secondly, data which were not obtained from the Child Health card were self-reported by the mother e.g. history of breastfeeding. To the extent that any mother failed to report accurately, either intentionally or unintentionally, our findings may have been biased. Furthermore, the 2000 Iraqi MICS did not collect data on child mortality. This, therefore, means that only children who were alive during the period of survey were included in the survey. Previous reports have indicated that child mortality was much higher during the period 1991 to 2003 than previously. The extent of survivor bias is therefore unknown but may have occurred i.e. children who were more likely to have diarrhoea or respiratory illness or both may have been more likely to have died and therefore missed out from this study.

## Conclusion

In a study of under-five children in Iraq in 2000, we found that history of ARI and diarrhoea were negatively associated with lower socio-economic status, adequate disposal of children' stool and dirty water but the results were inconsistent in terms of access to potable water and sanitation facilities due to the non-functioning of water and sewage plants after the war. Improvements in water quality and sanitation are vital in the reduction of diarrhoeal diseases. Further research focussing on risk factors for diarrhoea and acute respiratory infections in the context of post war Iraq should be conducted.

## Competing interests

The authors declare that they have no competing interests.

## Authors' contributions

SS design data analysis plan, conducted data analysis and participated in drafting of manuscript. ASM acquired data from UNICEF, participated in the interpretation of results and led the manuscript drafting effort. ER participated in the interpretation of the results and drafting of the manuscript.
